# Diseases from the Immoderate Use of Tobacco

**Published:** 1847-07

**Authors:** Thomas Laycock


### SELECTIONS

FROM

### AMERICAN AND FOREIGN JOURNALS.

*On the Diseases resulting from the immoderate Use of Tobacco.* By THOMAS LAYCOCK.—The consequences of smoking tobacco are manifested in the buccal and pharyngeal mucous membrane, and their diverticular; on the stomach, the lungs, and the heart, and on the brain and nervous system. With regard to these consequences, it may be generally stated here that they vary according to the quantity of tobacco smoked, and according to the pathological conditions and peculiarities of the individual himself. Some persons will smoke a very large quantity before certain symptoms arise, while others experience these with a very small quantity. The amount consumed by habitual smokers varies from half an ounce to twelve ounces per week. The usual quantity is from two to three ounces. Inveterate cigar smokers will consume from four to five dozen per week of the lighter kind of cigars, as Manillas, Bengal cheroots, etc.

The first and simplest morbid result of excessive smoking is an inflammatory condition of the mucous membrane of the lip and tongue, and this sometimes ends in a separation of the epithelium. Then the tonsils and pharynx suffer, the mucous membrane becoming dry and congested. If the throat be examined it will be observed to be slightly swollen, with congested veins meandering over the surface, and here and there a streak of mucus. The inflammatory action also extends upwards into the posterior nares, and the smoker feels from time to time a discharge of mucus from the upper part of the pharynx, in consequence of the secretion from the mucous membrane of the nares collecting within them. Sometimes the anterior nares suffer, but in this case the irritation is not marked by increased secretion so much as by tickling and itching within them. The irritation will also pass to the conjunctiva (and I am inclined to think from the nares, and not by the direct application of smoke to the eye), and the results are heat, slight redness, lachrymation, and a peculiar spasmodic action of the orbicularis muscle of the eye, experienced, together with intolerance of light, on awaking from sleep in the morning.

I think the frontal sinuses do not escape, for I find that one of the symptoms very constantly experienced after excessive smoking is a heavy dull ache precisely in the region of these sinuses. But, descending along the alimentary canal, we come to the stomach, and here we find the results to be, in extreme cases, the symptoms of gastritis. There is pain and tenderness on pressure of the epigastrium, anorexia, nausea on taking food, and constant sensation of sickliness and desire to expectorate.

The action of the heart and lungs is impaired by the influence of the narcotic on the nervous system, but a morbid state of the larynx, trachea, and lungs, results from the direct action of the smoke. The voice is observed to be rendered hoarser, and with a deeper tone; sometimes a short cough results; and in one case that came under my notice ulceration of the cartilages of the larynx was, I felt quite certain, a consequence of excessive use of tobacco. This individual had originaly contracted the habit of smoking when a sailor, and it had become so inveterate that he literally was never without a pipe in his mouth except when eating or sleeping. If he awoke in the night he lighted his pipe; the moment he finished a meal he did the same. It is only in extreme cases like this that the inference can be fairly made as to the morbid results of the habit, because there are so many other causes of disease to be estimated at the same time. This particular instance has, however during my experience, been corrobated by others of a like kind, and I have come to the conclusion that inflammation and ulceration of the larynx in men are almost exclusively peculiar to the slaves of excessive tobacco-smoking.

Hæmoptoë is another morbid condition distinctly traceable to this habit. The patient experiences a slight tickling low down in the pharynx or trachea, and hawks up rather than coughs up a dark grumous-looking blood. I have not been able to ascertain whence this comes. I have known it to flow out of the patient's mouth during the night, or to be effused shortly after lying down. It is a symptom worthy especial notice, however, because it gives great alarm, and may be readily mistaken for pulmonary hæmoptysis, or an expectoration of blood.

The action of tobacco-smoking on the heart, so far as I have observed, is depressing. The individual who, from some peculiarity of constitution, feels it in this organ rather than elsewhere, usually complains of a peculiar uneasy sensation about the left nipple—a distressing feeling—not amounting to faintness, but allied to it. In such an example no morbid sound can be detected, but the action of the heart is observed to be feeble, and slightly irregular in rhythm; yet not always so in the same person. An uneasy feeling is also experienced in or beneath the pectoral muscles, but oftener, I think, on the right side than on the left.

On the brain the action of tobacco-smoking is sedative. It appears to diminish the rapidity of cerebral action and check the flow of ideas through the mind. This, I think, is a certain result; and it is in consequence of this action that smoking is so habitual with studious men, or men of contemplative minds. The phrases, "a quiet pipe," or a "comfortable cigar," are significant of this sedative action. It differs, however, in kind from that of opium or henbane, because, as a *general* rule, tobacco does not dispose to sleep; it may in *individual* instances, but not generally, with tobacco-smokers. On the contrary, it rather excites to watchfulness, and in this respect is allied to green tea in its action: or, if not to wakefulness, to dreams, which leave no impression on the memory. When this effect has passed off, there appears to be a greater susceptibility in the nervous centres to impressions, as indicated by trembling of the hands, and irritability of temper.

There are a few facts which I would now state generally, and which appear as secondary results of smoking. Constipation and hæmorrhoids are often experienced by inveterate smokers. Acne of the face I have observed to be excited and kept up by the habit, and to disappear with the discontinuance of the latter. Blackness of the teeth and gum-boils are not uncommon results. There is also a sallow paleness of the complexion, an irresoluteness of disposition, a want of life and energy, to be observed ocaasionally in inveterate smokers, who are content with smoking,—that is to say, who do not drink. I have suspected also that it has induced pulmonary phthisis. It is thought that the sexual energy is impaired by the habit, but on this point I have no facts to detail. *Lon. Med. Gazette.*

